# The Blind Spot in Cardiogenic Shock: Patients Beyond the Current Paradigm

**DOI:** 10.1016/j.jscai.2026.105450

**Published:** 2026-05-22

**Authors:** Giulia Botti, Mirvat Alasnag, Alaide Chieffo

**Affiliations:** a Università Vita-Salute San Raffaele, Milan, Italy; b IRCCS Ospedale San Raffaele, Milan, Italy; c King Fahd Armed Forces Hospital, Jeddah, Saudi Arabia

**Keywords:** mechanical circulatory support, non-ST-elevation myocardial infarction, sex differences, ST-elevation myocardial infarction

Cardiogenic shock remains the most lethal complication of acute myocardial infarction, yet the contemporary evidence base has been mainly built around the patient with ST-elevation myocardial infarction (STEMI) with a clearly identifiable culprit lesion and a trajectory that prompts rapid revascularization and straightforward activation of advanced shock therapies. Women with non-ST-segment-elevation myocardial infarction complicated by cardiogenic shock (NSTEMI-CS), however, do not conform to this paradigm.

In this issue of *JSCAI*, Jain et al^1^ address this neglected intersection of sex, infarct phenotype, and shock severity. Using the United States Collaborative Network of TriNetX, the authors identified 58,524 adults with NSTEMI-CS between 2010 and 2025, of whom 21,872 were women. After 1:1 propensity matching, each sex cohort included 19,957 patients. Women had higher 30-day all-cause mortality, more major bleeding, and lower mechanical circulatory support (MCS) utilization while experiencing lower rates of recurrent heart failure exacerbation; recurrent myocardial infarction, stroke, vascular complications, and readmissions were otherwise similar. Although statistically significant, the absolute differences were small −1.3% for 30-day mortality and −2.1% for MCS use.

In a dataset of this size, such modest effects can readily reach statistical significance, so their clinical relevance should be interpreted with appropriate caution, as underlined by the authors. The stratified analyses highlighted that the disparity in MCS use appeared concentrated among patients who did not undergo percutaneous coronary intervention (PCI) and among those with baseline heart failure, whereas sex differences in mortality persisted across study eras. Notably, in patients without pre-existing heart failure, MCS utilization was similar between women and men. This suggests that the observed overall disparity may be driven, at least in part, by women with chronic heart failure presenting with NSTEMI-CS, and therefore not due to a primarily ischemic etiology. Overall, the study places women at the center of a clinical phenotype in which they may be disproportionately represented yet remain insufficiently studied (Figure 1).

**Figure 1:**
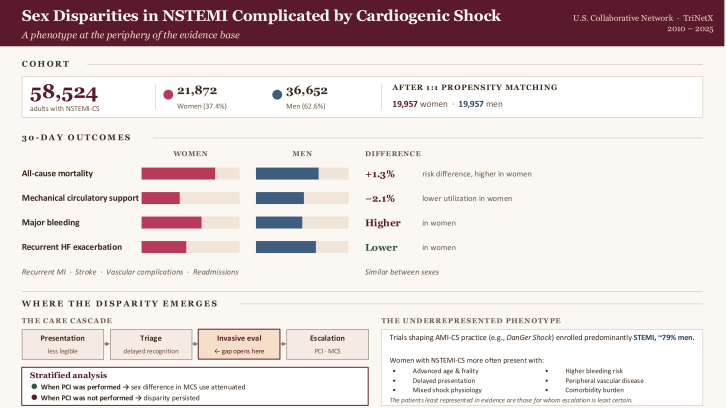
**Sex disparities in NSTEMI complicated by cardiogenic shock.** AMI-CS, acute myocardial infarction-cardiogenic shock; HF, heart failure; MCS, mechanical circulatory support; NSTEMI-CS, non–ST-elevation myocardial infarction-cardiogenic shock; PCI, percutaneous coronary intervention.

In this perspective, efforts such as the recent Society for Cardiovascular Angiography & Interventions/European Association of Percutaneous Cardiovascular Interventions/Association for Acute Cardiovascular Care expert consensus statement on cardiogenic shock in women aim not only to synthesize the available evidence and provide practical guidance but also to clearly identify existing gaps in knowledge.^2^

Women with acute myocardial infarction are more likely than men to present with NSTEMI and are therefore more frequently subject to delays to PCI and are less often transferred to PCI-capable facilities.^3^ Moreover, not all NSTEMI-CS is driven by obstructive coronary disease. Women are more frequently affected by myocardial infarction with nonobstructive coronary artery disease (MINOCA), which does not result in PCI but may be complicated by cardiogenic shock.^4^ Although generally characterized by better hemodynamic profiles than obstructive MI, the prevalence and progression of MINOCA are still poorly characterized and is being investigated in ongoing registries.^5^ Overall, NSTEMI-CS is often less immediately legible than STEMI-CS, the culprit anatomy may be less obvious, ischemic burden may accumulate before diagnosis, and clinical deterioration may be attributed to age, frailty, and decompensated chronic disease rather than acute coronary hypoperfusion. In shock, such delays exponentially impact outcomes: a syndrome defined by time-sensitive hemodynamic collapse becomes even harder to treat when it is recognized late and interpreted through a less urgent clinical lens.^6^

The strengths of the analysis by Jain et al are substantial. The cohort is large, contemporary, and drawn from routine practice rather than a highly selected trial population, and the conclusion is measured: women had lower MCS use and slightly higher mortality, but whether one explains the other cannot be determined from the available data.

The study’s limitations are as instructive as its findings.^1^ Administrative datasets lack the granularity to capture shock severity, metabolic derangement, vasopressor requirements, invasive hemodynamics, coronary anatomy, infarct size, and the timing and rationale behind revascularization or MCS use. However, these are precisely the factors that determine whether decisions reflect appropriate individualized care or disparities in escalation; without a detailed characterization of shock phenotypes, it remains difficult to determine potential drivers of disparity beyond sex. Nevertheless, across ischemic CS cohorts, women have repeatedly been shown to receive less invasive management, including lower rates of coronary angiography, PCI, and temporary MCS, despite a similar degree of severity.^7^

In this perspective, the signal around PCI in the study by Jain et al deserves to be outlined. Once a patient underwent PCI, the sex difference in MCS use appeared to attenuate; when PCI was not performed, the disparity persisted. This suggests that the central inequity may emerge not at the level of device preference, but earlier—during triage, invasive evaluation, shock team activation, or candidacy assessment.

Much of contemporary AMI-CS practice has been shaped by trials dominated by STEMI and disproportionately male patients. Most recently, the DanGer Shock trial represented a major advance, demonstrating improved survival with microaxial flow pump support in selected patients with STEMI-related shock.^8^ However, because it enrolled a highly selected, predominantly STEMI population that was 79% male patients, it also highlights the gap between trial-defined shock and real-world heterogeneity of ischemic CS. Patients with advanced age, peripheral vascular disease, bleeding risk, frailty, delayed presentation, or mixed shock physiology are less likely to be enrolled in invasive support trials. Those same features are overrepresented in women with NSTEMI-CS.^2^ Thus, the patients for whom clinicians feel least certain about escalation are often those least represented in the evidence invoked to justify or withhold it.

Moreover, shock teams should analyze whether their activation criteria unintentionally privilege the most dramatic presentations while missing the slower, less obvious, but equally dangerous trajectory of NSTEMI-CS in older women. Programs should further examine whether women are less likely to be transferred to tertiary shock centers or to undergo pulmonary artery catheterization or PCI.

Registry and real-world data should move beyond binary reporting to dissect NSTEMI-CS as a distinct phenotype, capturing diagnostic delays, comorbidity burden, revascularization strategies, and differential responses to support. Equally important is understanding not just whether MCS is used, but why it is withheld—be it vascular access constraints, frailty, neurological status, perceived futility, or late presentation. Without this granularity, the field will continue to conflate biology, clinical judgment, and inequity without the means to disentangle them.

Equity in shock care will not be achieved by equalizing device use alone. It requires re-engineering clinical pathways so that recognition, invasive evaluation, and escalation are driven by objective physiology rather than by resemblance to historical trial populations. Jain et al deserve credit for bringing this gap into focus. Their work does not resolve whether lower MCS use underlies excess mortality in women with NSTEMI-CS, nor does it establish that more aggressive support would improve outcomes. However, it underscores that women with NSTEMI-CS remain peripheral to the evidence base. If our systems function optimally only for those who mirror prior trial cohorts, the issue is not merely underrepresentation—it is that our definition of the “typical” shock patient is no longer fit for contemporary practice.

## CRediT authorship contribution statement

**Giulia Botti:** Conceptualization, Writing – original draft. **Mirvat Alasnag:** Conceptualization, Writing – original draft, Writing – review & editing. **Alaide Chieffo:** Conceptualization, Writing – review & editing.

## Declaration of competing interest

Alaide Chieffo is a speaker and/or receives consulting fees from Abbott Vascular, Johnson & Johnson MedTech, Cordis, Boehringer Ingelheim, Menarini, and Penumbra. Giulia Botti and Mirvat Alasnag reported no financial interests.
